# Left Ventricular Assist Device Implantation Combined with Bentall Procedure

**DOI:** 10.21470/1678-9741-2018-0123

**Published:** 2019

**Authors:** Safa Gode, Korhan Erkanlı, Serdar Başgoze, Selahattin Turen, Meliha Zeynep Kahraman

**Affiliations:** 1 Departments of Cardiovascular Surgery, Istanbul Mehmet Akif Ersoy Thoracic and Cardiovascular Surgery Training and Research Hospital, İstanbul, Turkey.; 2 Departments of Cardiology, Istanbul Mehmet Akif Ersoy Thoracic and Cardiovascular Surgery Training and Research Hospital, İstanbul, Turkey.; 3 Departments of Anesthesiology, Istanbul Mehmet Akif Ersoy Thoracic and Cardiovascular Surgery Training and Research Hospital, İstanbul, Turkey.

**Keywords:** Heart-Assist Devices, Aortic Valve Insufficiency - Surgery, Treatment Outcome, Case Reports

## Abstract

Ventricular assist devices (VADs) are an important technological development for patients with end-stage heart failure, and approximately 50% of these patients require various additional cardiac procedures. Here we presente the case of a patient suffering from severe aortic insufficiency, aortic root dilatation, and an ascending aortic aneurysm with end-stage decompensated heart failure. We performed the Bentall procedure combined with a left VAD implantation during the same session. The postoperative period was uneventful for this patient, and he was discharged on the 32nd postoperative day. The heart failure symptoms of the patient are reasonable, and he is still on the heart transplantation waiting list.

**Table:** 

Abbreviations, acronyms & symbols	
CPB	= Cardiopulmonary by-pass
CVP	= Central venous pressure
LV	= Left ventricle
LVAD	= Left ventricular assist devices
NYHA	= New York Heart Association
RV	= Right ventricle
RVSWI	= Right ventricular stroke work index
TAPSE	= Tricuspid annular plane systolic excursion
TPG	= Transpulmonary gradiente
VADs	= Ventricular assist devices

## INTRODUCTION

Ventricular assist devices (VADs) are an important technological development for patients with decompensated end-stage heart failure^[1]^. Unfortunately, approximately 50% of the patients with end-stage heart failure require additional cardiac surgical procedures. Rarely have we presented a case of a patient whose Bentall procedure was combined with a left VAD (LVAD) implantation during the same session.

## CASE REPORT

A 46-year-old male patient with dilated cardiomyopathy was admitted to our clinic with New York Heart Association (NYHA) class 3-4 functional capacity.

**Fig. 1:**
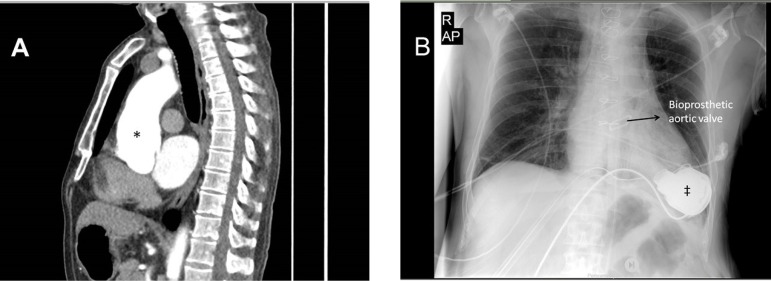
A)Preoperative computerized tomography imaging, B) Postoperative Chest X-Ray. *Ascending aortic aneurysm, left ventricular assist device

Physical examination revealed that the heart rate was 90 beats/min, blood pressure was 105/65 mmHg, and respiratory rate of 26 breaths per minute. There was crepitation on bilaterally basal segments of lungs and 3/6 diastolic murmur was present in the aortic valve area with auscultation. There was no pathological finding in other systems. Despite maximal medical treatment and intra-aortic balloon pumping, hemodynamic deterioration developed in the second day of hospitalization. Systolic blood pressure was 85 mmHg, signs of organ malperfusion with altered mental status; cold, clammy skin; oliguria (30 ml/h); increased serum-lactate (8 mEq/L). Echocardiography revealed that left ventricular (LV) ejection fraction was 20%, and he presented with severe aortic insufficiency, moderate calcific aortic stenosis, aortic annular dilatation and an ascending aortic aneurysm. His central venous pressure (CVP) was 13 mmHg, alanine transaminase was 48 IU/l, aspartate transaminase was 59 IU/l, international normalized ratio was 1.27, tricuspid annular plane systolic excursion (TAPSE) was 14 mm, pulmonary vascular resistance was 2.4 Wood Units, transpulmonary gradient (TPG) was 8 mmHg, and right ventricular stroke work index (RVSWI) was 370 mmHg*ml/m^2^. Therefore, no pulmonary vasodilatatory agent, including nitroprusside or milrinone, was required for the functional reduction of the RV. The ascending, arcus, and descending aortic diameters were 55 mm, 35 mm, and 33 mm, respectively, in the computed tomographic imaging (Figure 1).

This patient was on the heart transplantation list, but due to the progression of his clinical status and hemodynamic deterioration, our heart team decided to perform an LVAD implantation and Bentall procedure.

### Surgical Procedure

After conducting a median sternotomy, the pericardium was opened and the aorta was assessed. Sinus valsalva and proximal segment of ascending aorta were aneurysmatic and the aortic tissue was very thin and fragile. There was normal size aorta segment on the distal ascending aorta for cannulation and cross clamping. Therefore, an aortic arterial cannulation was performed at the level of brachiocephalic artery and a right atrial venous cannulation was also performed. Cardiopulmonary bypass (CPB) was established in the standard fashion. The patient's body was cooled to 28°C. After applying an ascending aortic cross clamp, an aortotomy was performed on the aneurysmal ascending aorta, and cold blood cardioplegia was delivered to the coronary arteries via the coronary ostia. There was commissural fusion and diffuse calcification on the aortic annulus, and the aortic valve exhibited a tricuspid structure. It was excised, and the coronary ostia were prepared as buttons. The ascending aortic aneurysmal tissue was also excised, and a distal aortic anastomosis area was prepared. A conduit with a no. 28 Dacron tube graft and a no. 25 Carpentier-Edwards Perımount pericardial aortic bioprosthesis valve was placed into the aortic annulus with the aid of pledgets. Then, the coronary buttons were anastomosed to the Dacron graft, and a distal aortic anastomosis was performed. Thus, the Bentall procedure was completed.

**Fig. 2:**
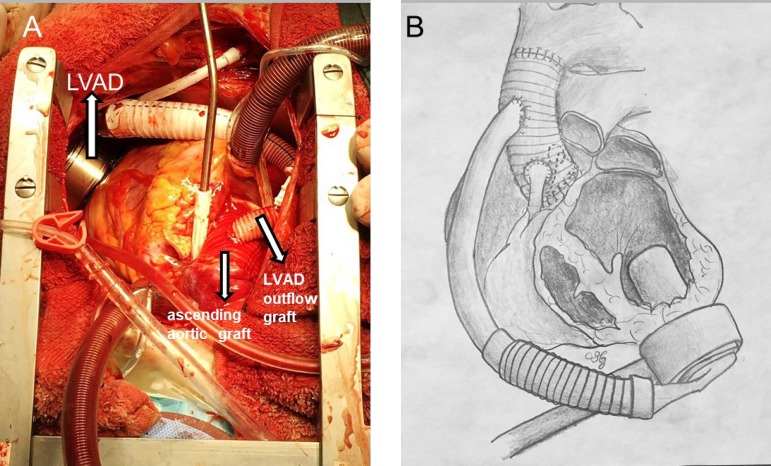
A) Peroperative Picture, B) Operation Drawing.

Next, the cross-clamp was removed, and the body was warmed to 36°C. The LV position was adjusted, and a suitable area on the LV was determined via transesophageal echocardiography for the LVAD implantation. The ring of a HeartMate 3 LVAD (Abbott Laboratories, Chicago, IL, USA) was implanted on the apex of the heart enforced with Teflon felt under beating heart conditions. The inflow cannula of the device was placed into the ventricular cavity, and the battery cable of the device was removed from the right inferior quadrant of the abdomen via the subxiphoid tunnel. All the air was evacuated from inside the device and heart, and the outflow graft of the device was anastomosed to the ascending aortic graft in an end-to-side fashion (Figure 2). Then, the device was started. While the support of the device increased, the support of the CPB was slowly decreased and ended. The last control of the device was performed via transesophageal echocardiography. The cardiac index was greater than 2.8 l/min/m^2^ at a speed of 8,400 rpm. The RV function was satisfactory: TAPSE=16 mm, TPG=10 mmHg, and RVSWI=405 mmHg*ml/m^2^. The patient was admitted to the intensive care unit in a stable condition. The CPB time was 138 minutes, and the aortic cross clamp time was 64 minutes.

The mean arterial pressure, CVP, cardiac rhythm, and pulmonary capillary wedge pressure were 75 mmHg, 14 mmHg, 83 beats/minute, and 10 mmHg, respectively. Therefore, the hemodynamic parameters were normal. The patient awoke 6 hours after the surgery, and his neurological status was entirely normal. The total drainage was 1,250 cc during the first day and 650 cc during the second day postoperatively. Three units of erythrocyte suspension were used in total. The extubation was performed at the postoperative 21st hour. RV failure findings were not observed, and the early postoperative period was satisfactory. This patient was discharged on postoperative day 32. The control examination was satisfactory in terms of the clinical, laboratory, and echocardiographic measurements during second month after the surgery (Figure 1). He is still on the heart transplantation list.

## DİSCUSSİON

In this case, we performed Bentall procedure combined with a LVAD implantation urgently. The patient was in the heart transplantation list. Hemodynamic and clinical deterioration occurred and maximal medical treatment and intra-aortic balloon pump were performed. But hemodynamic and clinical situation were not satisfactory. Ideal intervention was heart transplantation and ascending aorta replacement. But we did not have a chance to find an emergency donor for this patient with acute decompansated heart failure. Therefore, our hear team decided to perform an LVAD implantation and a Bentall procedure in the same session to bridge for transplantation.

When compared with other cardiac operations, patients with LVADs have a 6%-8% increased risk of requiring additional surgery within one year of the implantation^[2]^. Our patient with end-stage decompensated heart failure suffered from severe aortic insufficiency, aortic root and sinus valsalva dilatation and an ascending aortic aneurysm. Therefore, we performed the Bentall procedure combined with an LVAD implantation in the same session. Takeda et al.^[3]^ reported a case with multiple saccular ascending aortic aneurysms and end-stage heart failure, and they also successfully performed an aortic surgery combined with an LVAD implantation. Moreover, the effects of a continuous-flow LVAD on aortic wall stress were studied by Segura et al.^[4]^. They evaluated the ascending aortic tissue before and after the implantation of an LVAD by comparing the medial degeneration, elastic fiber fragmentation, medial fibrosis, and atherosclerosis. They observed that the LVAD had a significant effect on the ascending aorta. Thus, surgical interventions for ascending aortic aneurysms seem unavoidable in these patients. Therefore, our cardiac team decided to conduct the Bentall procedure combined with an LVAD implantation in our patient.

Patients with ascending aortic aneurysms have further risks during LVAD implantation procedures. For example, a rupture may occur during the outflow graft anastomosis due to the fragile and weak ascending aortic tissue. Therefore, changing the aneurysmal ascending aorta is necessary before the LVAD implantation, even though this increases the surgical risk. The other choices for improving aortic valve insufficiency at the time of an LVAD implantation are an aortic valve repair or closure. Although there is no clear superiority between these methods in the literature, Robertson et al.^[5]^ determined that an aortic valve closure significantly increases mortality. Therefore, we chose the aortic valve replacement method to avoid the increased risk of mortality in our case.

In summary, a rare operation that combined the Bentall procedure with an LVAD implantation was performed successfully, and the patient is now waiting for a heart transplantation in a more comfortable state.

**Table:** 

Authors' roles & responsibilities	
SG	Substantial contributions to the conception or design of the work; or the acquisition, analysis, or interpretation of data for the work; drafting the work or revising it critically for important intellectual content; final approval of the version to be published
KE	Substantial contributions to the conception or design of the work; or the acquisition, analysis, or interpretation of data for the work; final approval of the version to be published
SB	Drafting the work or revising it critically for important intellectual content; final approval of the version to be published
ST	Drafting the work or revising it critically for important intellectual content; final approval of the version to be published
MZK	Drafting the work or revising it critically for important intellectual content; final approval of the version to be published
